# Identification and clinical evaluation of diagnostic biomarkers for ischemic cardiomyopathy based on machine learning and transcriptomics

**DOI:** 10.3389/fcvm.2026.1838385

**Published:** 2026-08-07

**Authors:** Xin Zhang, Shaohua Cao, Wangwang Duan, Jianlin Huang

**Affiliations:** 1 Department of Pharmacy, Yanan University Affiliated Hospital, Yan'an, Shaanxi, China; 2 School of Nursing and Health, Xi'an Innovation College of Yan'an University, Xi'an, Shaanxi, China

**Keywords:** DEG, GEO, ischemic cardiomyopathy, machine learning, WGCNA

## Abstract

**Background:**

Ischemic cardiomyopathy (ICM) is a leading cause of heart failure, yet precise molecular tools for potential diagnosis remain limited. This study aims to identify and clinically evaluate novel diagnostic biomarkers for ICM by integrating comprehensive transcriptomic analysis, machine learning algorithms, and real-world serological assessment.

**Methods:**

Gene expression profiles from the GEO database were systematically analyzed using weighted gene co-expression network analysis (WGCNA) and 12 distinct machine learning algorithms to screen for optimal diagnostic targets. Crucially, to bridge the gap between computational prediction and clinical application, the identified core diagnostic genes were evaluated at the protein level using an independent clinical cohort. Peripheral serum samples from 90 individuals (45 ICM patients and 45 controls) were analyzed via enzyme-linked immunosorbent assay (ELISA).

**Results:**

We identified 501 differentially expressed genes, with the MEcyan WGCNA module showing the strongest correlation with ICM. Among the 12 evaluated machine learning models, AdaBoost showed the highest internal predictive performance (AUC = 0.976). However, this estimate is exploratory and potentially optimistic due to feature pre-selection prior to cross-validation. From this candidate pool, SEPP1, CILP, and FRZB were selected *post hoc* as the core diagnostic targets based on their univariate performance and clinical translational suitability. These markers were significantly associated with complement activation, extracellular matrix remodeling, and immune cell infiltration. Most importantly, our clinical ELISA experiments provided preliminary protein-level evidence that the serum protein levels of SEPP1, CILP, and FRZB were significantly elevated in ICM patients compared to controls, which is consistent with the bioinformatic predictions.

**Conclusion:**

By integrating computational screening with preliminary clinical serological analysis, this study identifies SEPP1, CILP, and FRZB as potential serological biomarkers for ICM. This computational model and the accompanying preliminary serological evidence provide a theoretical basis for future clinical exploration and diagnostic biomarker development.

## 1 Introduction

Ischemic cardiomyopathy (ICM) is a significant cause of heart failure, resulting from inadequate blood supply to the myocardium due to coronary artery disease (1). This condition leads to structural and functional abnormalities in the heart, posing severe risks to patient health and increasing healthcare costs (2, 3). The potential diagnosis of ICM is critical for improving patient outcomes; however, current diagnostic modalities primarily rely on clinical imaging and serum biomarkers, which often lack sufficient accuracy in timely and precise diagnosis (4, 5). Moreover, there is a notable gap in effective molecular diagnostic tools that could enhance treatment strategies and prognostic evaluations, underscoring the necessity for innovative research into the molecular mechanisms underlying ICM.

Previous studies have identified various genes associated with the pathophysiology of ICM, yet a comprehensive analysis of gene expression patterns and their regulatory networks remains underexplored (6–8). The advent of systems biology approaches, including weighted gene co-expression network analysis (WGCNA), offers a promising avenue for dissecting these complex gene interactions (9). Additionally, the integration of machine learning algorithms for the identification of diagnostic biomarkers based on gene expression data presents a novel approach to enhance ICM diagnostics (10).

In this study, we utilized publicly available data from the Gene Expression Omnibus (GEO) database to perform differential expression analysis, followed by WGCNA to construct gene co-expression modules. Subsequently, we employed functional enrichment analyses, including Reactome and Gene Ontology (GO), to elucidate the biological pathways involved in ICM. The ultimate goal was to identify key diagnostic genes through various machine learning models and to develop a predictive diagnostic model based on these critical genes. This multifaceted approach aims to provide a robust framework for understanding the molecular landscape of ICM and its potential implications for precision medicine.

Furthermore, the interplay between immune cell infiltration and ICM has begun to attract attention, as immune responses are known to influence cardiac remodeling and progression of heart failure (11). Preliminary findings suggest that specific immune cell types may correlate with disease severity and patient outcomes (12, 13). Analyzing the immune microenvironment in conjunction with gene expression profiles could yield valuable insights into the immunological aspects of ICM, potentially leading to novel therapeutic strategies that target both the molecular and immunological facets of the disease (14–16).

Through this research, we aim to bridge the existing gaps in the molecular understanding of ICM and to advance the development of precise diagnostic tools. By leveraging advanced analytical methodologies and machine learning techniques, this study aspires to identify novel biomarkers that could facilitate potential diagnosis and improve therapeutic interventions for patients suffering from ICM.

## 2 Materials and methods

### 2.1 Data download and processing

Datasets GSE1869, GSE5406, and GSE26887 were retrieved from the GEO database (http://www.ncbi.nlm.nih.gov/geo) in December 2024 using the keywords (‘Ischemic Cardiomyopathy’ OR ‘ICM’) (17). To minimize batch effects and ensure anatomical consistency, we strictly included datasets utilizing Affymetrix microarray platforms (excluding RNA-seq datasets like GSE116250) and ensured all samples were derived exclusively from left ventricular myocardial tissues. The GSE1869 dataset includes 6 normal samples and 10 ICM samples; the GSE5406 (GPL96) dataset includes 16 normal samples and 108 ICM samples. These two datasets were merged and batch normalized using the “sva” package in R, and the merged dataset was used as the training set (18). The GSE26887 dataset contains 5 normal samples and 19 ICM samples, which served as the validation set.

### 2.2 Differential expression gene screening

DEGs for ICM were identified using the limma package in R. To capture subtle but topologically critical transcriptomic alterations characteristic of chronic cardiovascular remodeling for downstream network analysis, the screening criteria were set at |log2 fold change (FC)| > 0.3 and adjusted *p*-value < 0.05 (using the Benjamini-Hochberg method) (19). The “ggplot2” and “pheatmap” packages were utilized to create volcano plots and heatmaps (20, 21).

### 2.3 Weighted gene Co-expression network analysis (WGCNA)

WGCNA was applied to identify key modules associated with ICM (22). To minimize background noise, genes with a median absolute deviation (MAD) of less than 0.5 were excluded from downstream analyses. Subsequently, a power function was used to construct a weighted adjacency matrix, and the “pickSoftThreshold” function was applied to find the optimal soft threshold (*β*). The adjacency matrix was transformed into a topological overlap matrix (TOM). The gene ratio in the network was defined as the sum of adjacency relationships between a gene and all other genes, and the corresponding dissimilarity (1-TOM) was calculated. Third, genes with similar expression profiles were classified into gene modules using average linkage hierarchical clustering, with a minimum module size (*n* = 10) set to construct a gene dendrogram. Finally, to further analyze the modules, we calculated the dissimilarity of module feature genes, set the cutting line of the module dendrogram to 0.4, and merged some modules for further study.

### 2.4 Enrichment analysis

Reactome pathway analysis and Gene Ontology (GO) functional annotation were performed using the R package “clusterProfiler” (23, 24). The background gene list was defined as all valid transcripts detected in the merged microarray datasets. Pathways were filtered with a minimum gene set size of 10 and a maximum size of 500. A pathway was considered statistically significant if the Benjamini-Hochberg (BH) adjusted *p*-value was less than 0.05. A protein-protein interaction (PPI) network was constructed using the STRING database (https://cn.string-db.org/), with a minimum required interaction score set at 0.400 (medium confidence) (25).

### 2.5 Machine learning

To develop a model capable of predicting ICM, a series of advanced machine learning algorithms were employed. For systematic evaluation, these algorithms were classified into three main categories: (1) Ensemble and tree-based models, including Random Forest (RF), Gradient Boosting Machine (GBM), eXtreme Gradient Boosting (XGB), AdaBoost (ADA), Bagging (BAG), and Decision Tree (DT); (2) Regularized and linear models, including Generalized Linear Model (GLM) and Least Absolute Shrinkage and Selection Operator (LASSO); and (3) Classical classifiers, including Support Vector Machine (SVM), K-Nearest Neighbors (KNN), Neural Network (NNET), and Naive Bayes (NB) (26–30). To gain insights into the performance of these models, we utilized the DALEX package to visualize the residual distribution of the twelve methods. Through this analysis, we identified the most effective machine learning model, prioritizing the minimization of residuals. This approach led us to identify three key genes as important diagnostic biomarkers. Furthermore, to assess the accuracy of our model, we generated ROC curves using the “pROC” package in R and calculated the area under the curve (AUC) to quantify the predictive capability of the model (31).

### 2.6 Immune cell infiltration and correlation analysis

The CIBERSORT algorithm was used to evaluate the relative abundance of 22 lymphocyte subtypes in each normal and ICM sample (32). Subsequently, Spearman correlation analysis was performed to assess the relationship between key genes in ICM and immune cells.

### 2.7 Construction and validation of the nomogram model

A nomogram model was constructed using the “rms” package in R based on candidate key genes. The “score” for each candidate gene was represented by “points,” while the “total score” was the sum of the scores of all aforementioned genes. Additionally, calibration curves and decision curve analysis (DCA) were used to evaluate the predictive capability of the nomogram model (33).

### 2.8 Diagnostic value of central Key genes

The diagnostic value of the three selected genes was evaluated using univariate ROC curves. It is important to note that the locked multivariable AdaBoost model was not applied to the external dataset; rather, the GSE26887 dataset was used strictly for the univariate assessment of SEPP1, CILP, and FRZB.

### 2.9 Evaluation of peripheral blood samples

A total of 45 patients diagnosed with ischemic cardiomyopathy (ICM) and 45 controls were included from Yanan University Affiliated Hospital. The control group consisted of community individuals who underwent routine physical examinations at the hospital's Health Check-up Center, showing normal electrocardiograms, normal echocardiography, and specifically no clinical history or symptoms of coronary artery disease or heart failure. To strictly comply with ethical guidelines and avoid any prospective patient recruitment or additional invasive procedures, this study was based on the secondary use of residual, de-identified clinical serum samples left over from these routine laboratory tests. The sequential collection of these residual samples was conducted between February 2025 and November 2025, immediately following approval by the Ethics Committee of Yanan University Affiliated Hospital (Approval No. IIT-R-2024192). Consequently, the requirement for written informed consent was formally waived.

Serum samples (stored at −80 °C, ≤1 freeze-thaw cycle) were analyzed using ELISA kits (Shanghai Hengyuan Biological Technology Co., Ltd., Cat. NO. HB500-Hu, HB4266-Hu, and HB4268-Hu). Assays were performed in duplicate by personnel blinded to clinical assignments. Optical density was measured at 450 nm, and absolute concentrations were calculated using a four-parameter logistic (4-PL) curve-fit model in GraphPad Prism 9.0. The intra-assay and inter-assay coefficients of variation were <8% and <10%, respectively. Values below the limit of detection (LOD) were assigned the LOD value, while samples exceeding the quantification range were diluted and re-assayed.

### 2.10 Statistical analysis

All data processing and analyses were conducted using R software (version 4.2.2). The Wilcoxon test was used to assess statistical differences between two groups. Spearman correlation analysis was employed to examine the relationships between key genes and immune cells. A *p*-value of less than 0.05 was considered statistically significant. Where applicable, the reporting of this observational and predictive modeling study adheres to the principles of the STROBE and TRIPOD guidelines.

## 3 Results

### 3.1 Identification of DEGs between normal samples and ICM samples

**Figure 1:**
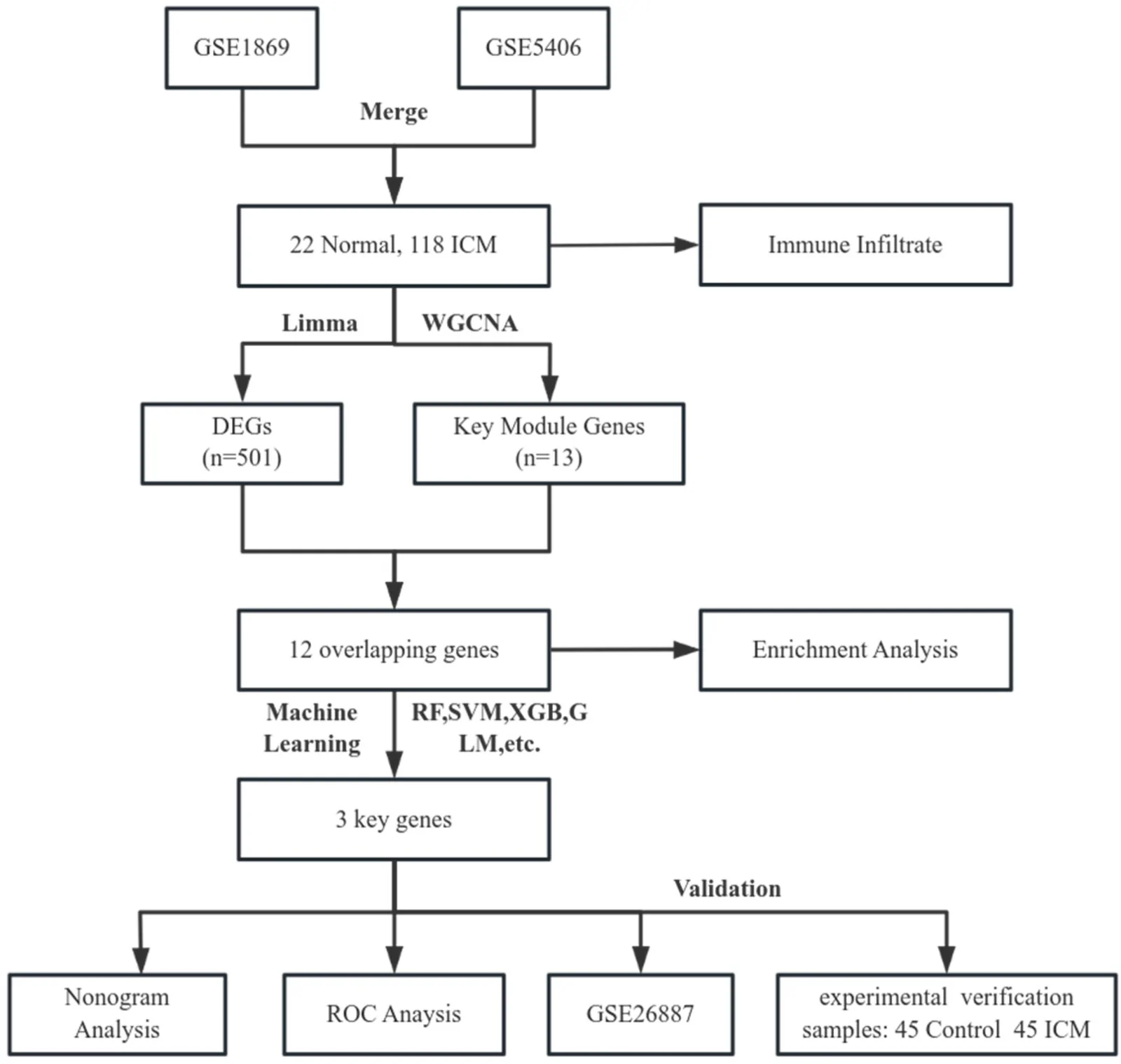
Work flowchart of this study.

**Figure 2:**
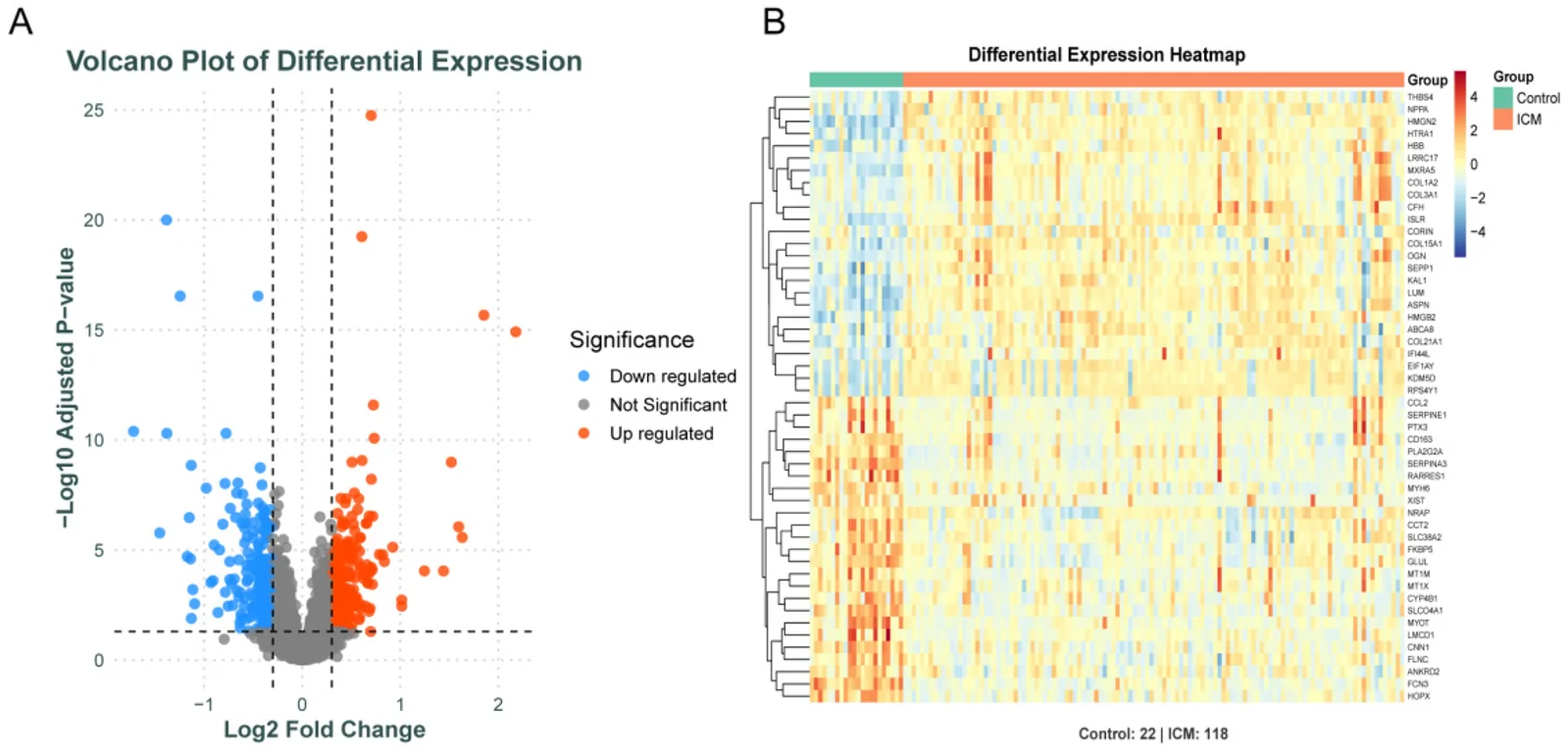
Screening DEGs of ICM. **(A)** The volcano plot of DEGs is shown when comparing ICM and normal samples. Downregulated genes are represented by blue circles, and upregulated genes are represented by red circles. **(B)** The heatmap shows the top 25 differentially expressed genes of ICM.

 Figure 1 illustrates the workflow of this study. Differentially expressed genes (DEGs) between normal and ICM samples were identified using the limma package. A total of 501 DEGs were screened, with 288 genes upregulated and 213 genes downregulated (Figure 2A). The heatmap displays the top 25 upregulated and downregulated DEGs (Figure 2B).

### 3.2 Construction of WGCNA and gene module screening

**Figure 3:**
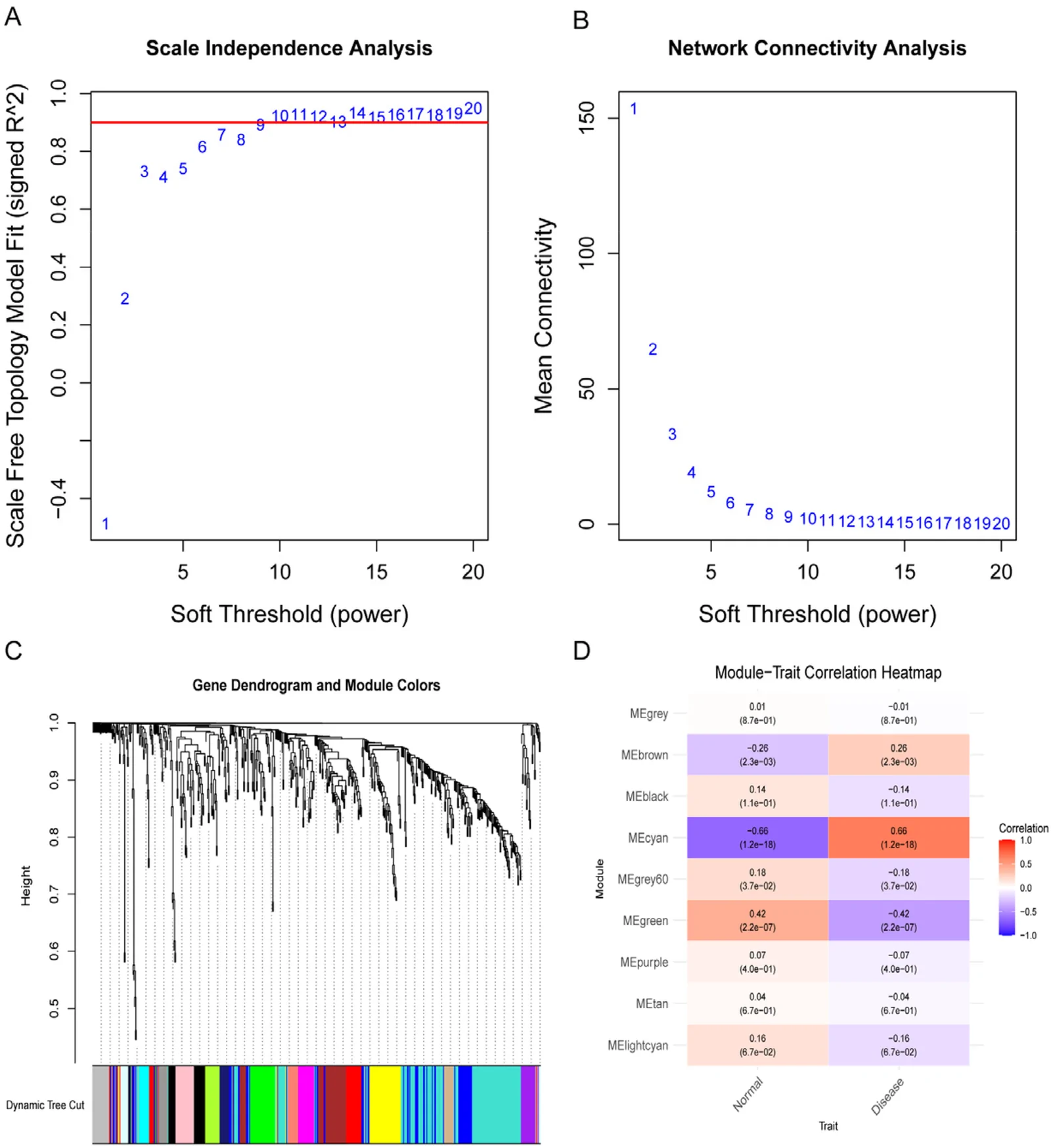
Identification of the most relevant module genes via WGCNA in ICM. **(A,B)** Based on the results of scale independence and average connectivity, *β*=7 was chosen as the optimal soft threshold. **(C)** Gene co-expression modules are displayed in different colors below the gene tree. **(D)** Heatmap of the association between modules and ICM. The cyan module shows a strong positive correlation with ICM. The correlation coefficient and *p*-value are represented by the numbers in parentheses.

The WGCNA algorithm was used to screen key gene modules closely related to ICM. A scale-free network was constructed using *β* = 7 and R^2^ = 0.9 as criteria (Figures 3A,B). A clustering dendrogram was created, and dynamic tree cutting was performed (Figure 3C), resulting in a total of 9 gene modules, which were presented in a heatmap (Figure 3D). The results indicated that the MEcyan module (co*r* = 0.66; *p* = 1.2e-18) was strongly positively correlated with ICM, while the MEgreen module (cor = −0.42; *p* = 2.2e-07) had a strong negative correlation with ICM. The MEcyan module contained 13 genes.

### 3.3 Functional enrichment analysis

**Figure 4:**
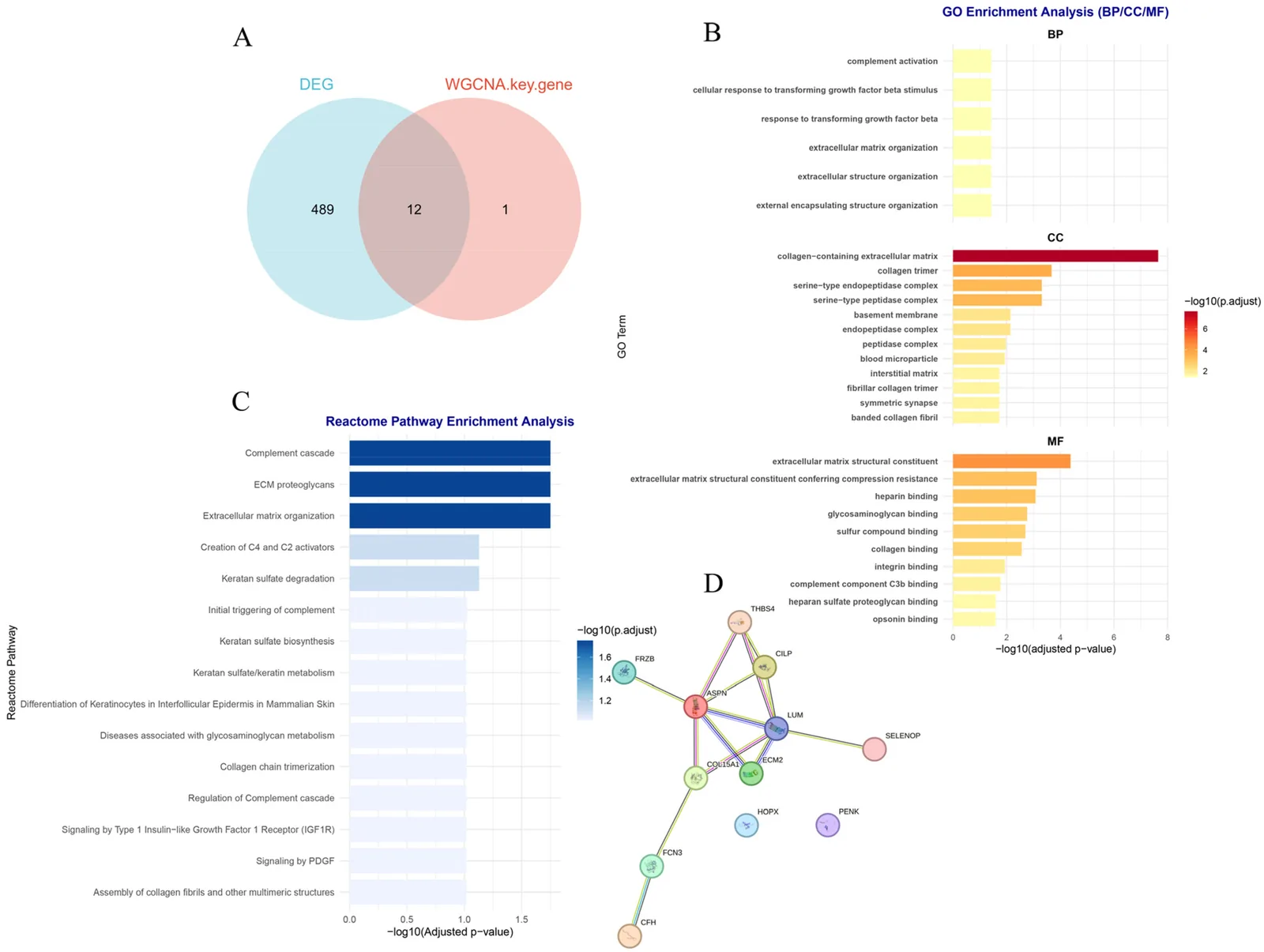
The functional enrichment and protein-protein interaction (PPI) analysis of these intersecting genes. **(A)** The Venn diagram shows that there are 12 intersecting genes in the DEGs of ICM and the WGCNA MEcyan module genes. **(B,C)** Gene ontology (GO) and Reactome Pathway Knowledgebase (Reactome) enrichment analysis of the 12 intersecting genes. **(D)** The PPI network shows the relationships between these genes.

There were 12 intersecting genes between the DEGs of ICM and the key module genes of ICM (Figure 4A), while one gene (CHRDL1) in the MEcyan module was excluded due to its marginal significance (*p* = 0.0605). The top four functional enrichment results indicated that these genes were primarily involved in complement activation, response to transforming growth factor beta (TGF-*β*) stimulus, response to TGF-*β*, and extracellular matrix organization (BP). They were also associated with collagen-containing extracellular matrix, collagen trimer, serine peptidase complex, basement membrane, and endopeptidase complex (CC), as well as extracellular matrix structural constituent, extracellular matrix structural constituent conferring compression resistance, heparin binding, glycosaminoglycan binding, and sulfur compound binding (MF) (Figure 4B). Reactome analysis demonstrated that the top three enriched pathways were complement cascade, ECM proteoglycans, and extracellular matrix organization (Figure 4C). These intersecting genes interacted within a protein-protein interaction (PPI) network, which constructed a network containing 12 nodes and 14 edges (Figure 4D).

### 3.4 Screening of core diagnostic genes using Various machine learning algorithms

**Figure 5:**
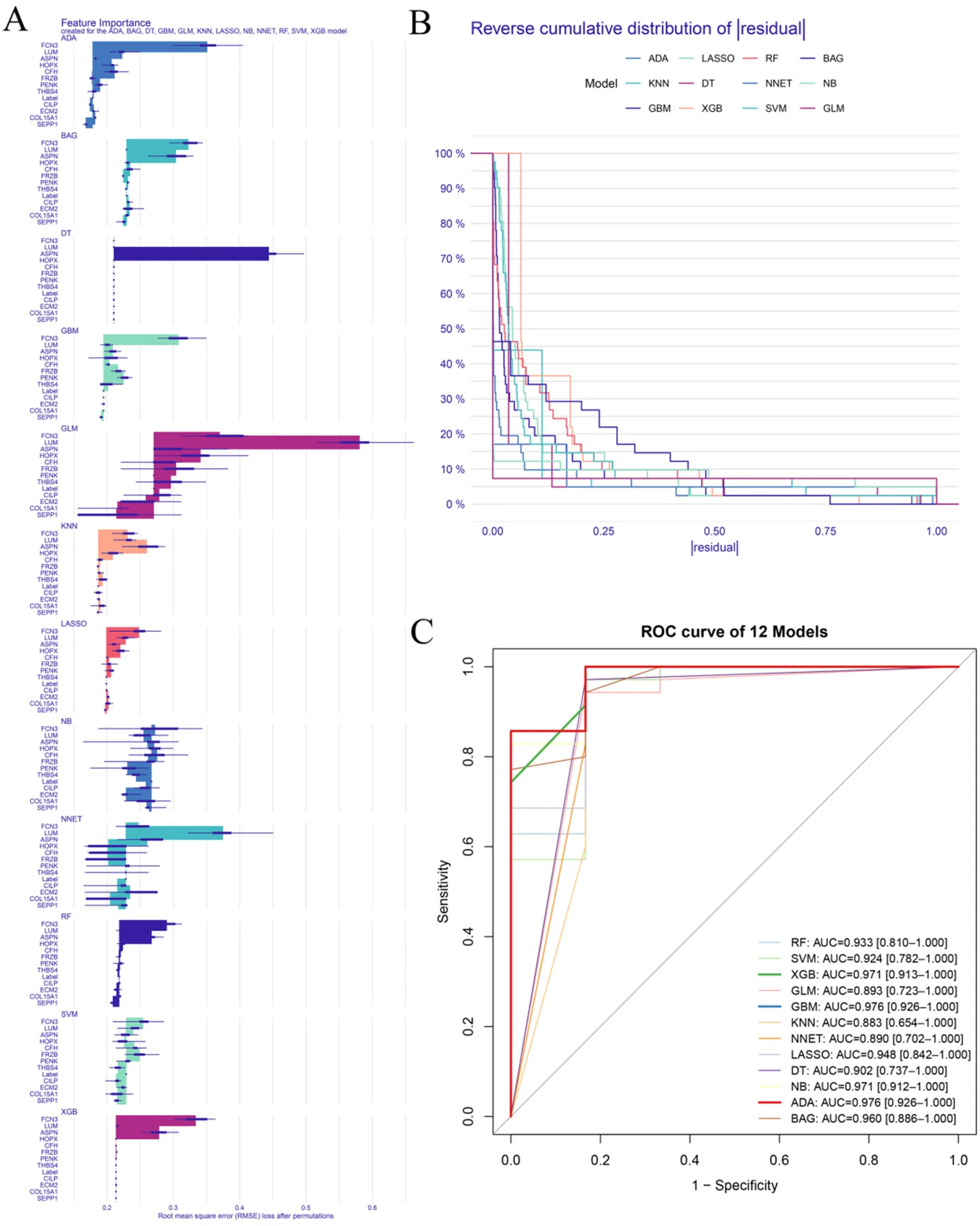
Evaluation of the twelve machine learning models. **(A)** Feature importance across the twelve algorithms based on Root Mean Square Error (RMSE) loss. “Label” denotes the clinical grouping variable used as an internal baseline. Error bars represent the standard deviation of the RMSE loss following feature permutations. **(B)** Cumulative residual distribution of the models. ROC curves of the twelve models. The top three performing models (ADA, GBM, and XGB) are highlighted with thicker lines. **(C)** Exploratory ROC curves of the twelve machine learning models. Note that the internal AUC estimates may be optimistic due to information leakage during feature selection. The top three performing models (ADA, GBM, and XGB) are highlighted with thicker lines.

To further construct a diagnostic model, twelve machine learning models (RF, SVM, XGB, GLM, GBM, KNN, NNET, LASSO, DT, NB, ADA, and BAG) were applied to screen high diagnostic value biomarkers based on 12 significant genes associated with ICM. Initially, we examined and compared the residual distribution of each model. The results indicated that the ADA model exhibited the lowest residuals compared to the other models (Figure 5B). Subsequently, we ranked the core diagnostic genes for each model based on root mean square error (RMSE) (Figure 5A). Importantly, to address the substantial class imbalance in the training dataset (118 ICM vs. 22 normal), we utilized stratified five-fold cross-validation to ensure that the proportional distribution of classes was strictly maintained across all folds. A fixed random seed [set.seed(123)] was applied prior to model training to ensure full reproducibility. Hyperparameters were optimized utilizing the default grid search strategies within the training folds. Notably, both the ADA and GBM models demonstrated the highest internal AUC values: ADA (AUC = 0.976) and GBM (AUC = 0.976) (Figure 5C). However, because feature pre-selection was conducted prior to cross-validation, these internal performance metrics should be regarded as exploratory and potentially optimistic. To provide a comprehensive comparison, the key model fit indices, including AUC values and residual metrics for all twelve algorithms, are summarized in Table 1. Considering both the minimal residual values and AUC values, we confirmed the superior performance of the ADA model in distinguishing patient populations. Based on the feature importance evaluations (RMSE loss upon permutation) in the DALEX explainer, genes such as FCN3 and LUM exhibited the highest dropout loss, indicating they were the most mathematically important features within the ADA ensemble model (Figure 5A). Importantly, SEPP1, CILP, and FRZB were not the top-ranked features in this machine-learning model. Instead, their downstream selection was a *post hoc* decision based on a combination of their robust univariate diagnostic performance and their translational suitability for standalone serological tests (ELISA). While top-ranked multivariate features often rely on complex collinear interactions, we prioritized SEPP1, CILP, and FRZB because their independent diagnostic potential makes them more practical for real-world clinical assessment.

**Table 1:** Performance comparison of the twelve machine learning models.

Model Category	Algorithm	AUC (95% CI)	RMSE
Ensemble & Tree-based	ADA	0.976 (0.926–1.000)	0.178
	GBM	0.976 (0.926–1.000)	0.195
	XGB	0.971 (0.913–1.000)	0.214
	RF	0.933 (0.810–1.000)	0.218
	BAG	0.960 (0.886–1.000)	0.229
	DT	0.902 (0.737–1.000)	0.210
Regularized & Linear	GLM	0.893 (0.723–1.000)	0.271
	LASSO	0.948 (0.842–1.000)	0.199
Classical Classifiers	SVM	0.924 (0.782–1.000)	0.229
	KNN	0.883 (0.654–1.000)	0.187
	NNET	0.890 (0.702–1.000)	0.228
	NB	0.971 (0.912–1.000)	0.267

AUC, area under the curve; CI, confidence interval; RMSE, root mean square error; ADA, AdaBoost; GBM, gradient boosting machine; XGB, eXtreme gradient boosting; RF, random forest; BAG, bagging; DT, decision tree; GLM, generalized linear model; LASSO, least absolute shrinkage and selection operator; SVM, support vector machine; KNN, K-nearest neighbors; NNET, neural network; NB, naive bayes.

### 3.5 Construction of the diagnostic model

**Figure 6:**
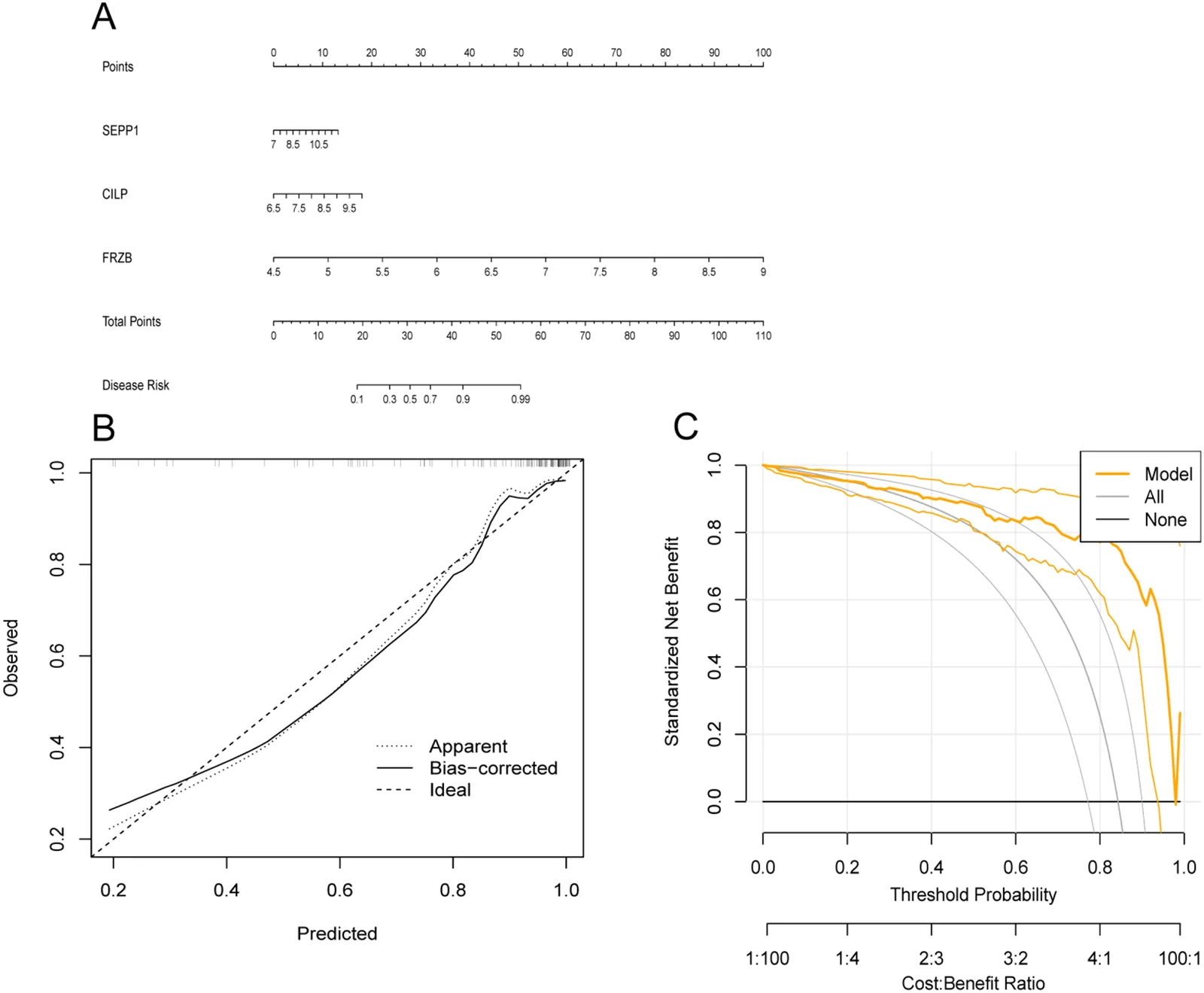
Construction of the nomogram. **(A)** The visual nomogram is used for diagnosing ICM. **(B)** The predictive accuracy of the nomogram model is assessed through the calibration curve. **(C)** DCA curve, used to evaluate the clinical applicability of the nomogram model. In the DCA plot, “All” assumes all patients are positive for the disease, “None” assumes no patients are positive, whereas “Model” represents the diagnostic strategy based on established gene signature.

We created a nomogram to determine the risk of developing ICM based on the three core diagnostic genes screened by the ADA model (Figure 6A). The calibration curve was used to assess the predictive efficiency of the nomogram model (Figure 6B). According to the calibration curve analysis, the error between actual and predicted ICM was minimal. Furthermore, the Decision curve analysis (DCA) results indicated that the nomogram exhibited a potential clinical net benefit within the transcriptomic cohort (Figure 6C).

### 3.6 Evaluation of diagnostic value

**Figure 7:**
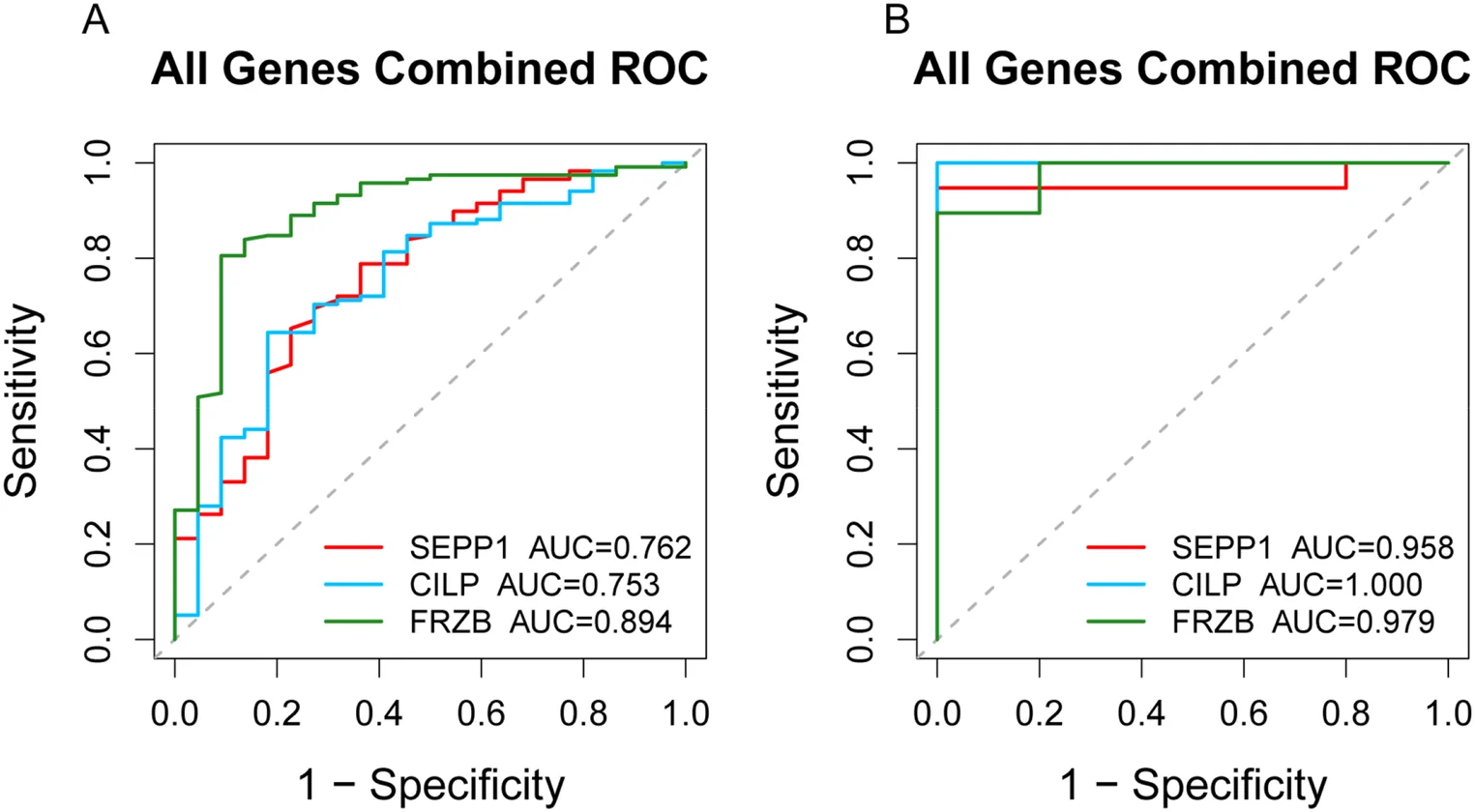
Evaluation of diagnostic value. **(A)** ROC curves for SEPP1, CILP, and FRZB in the training dataset. **(B)** ROC curves for SEPP1, CILP, and FRZB in the external validation dataset.

ROC curves were utilized to assess the diagnostic capabilities of the three core diagnostic genes in both the training dataset (Figure 7A) and the external validation dataset GSE26887 (Figure 7B). The detailed diagnostic metrics, including AUC values with 95% confidence intervals (CIs), sensitivity, specificity, and the threshold criteria (determined by Youden's index), are comprehensively summarized in Table 2. While the point estimates of AUC in the external dataset exceeded 0.95, these values exhibit notably wide 95% CIs, indicating a degree of statistical instability driven by the limited sample size.

**Table 2:** Detailed diagnostic performance metrics of the core diagnostic genes.

Gene	Dataset	AUC (95% CI)	Sensitivity (%)	Specificity (%)	Threshold
SEPP1	Training (GSE1869 + GSE5406)	0.762 (0.646–0.868)	65.3	77.3	9.822
	External (GSE26887)	0.958 (0.871–1.000)	94.7	80.0	7.629
CILP	Training (GSE1869 + GSE5406)	0.753 (0.632–0.856)	64.4	81.8	8.015
	External (GSE26887)	1.000 (1.000–1.000)*	100.0	100.0	8.206
FRZB	Training (GSE1869 + GSE5406)	0.894 (0.811–0.963)	80.5	90.9	5.625
	External (GSE26887)	0.979 (0.915–1.000)	100.0	80.0	5.820

* (Thresholds were determined based on the maximization of Youden’s J statistic. The extremely tight CI for CILP in the external dataset is a mathematical artifact of complete separation in a very small minority class, highlighting the instability of the estimate.).

### 3.7 Baseline characteristics of the clinical cohort

The baseline clinical characteristics of the 90 participants are summarized in Table 3. Age, sex, and body mass index (BMI) were well-matched between the two groups. As expected in a real-world clinical setting, the ICM group had a higher prevalence of hypertension, a higher rate of statin usage, and altered cardiac/renal function indices compared to controls (all *p* < 0.05). To account for these baseline differences, an Analysis of Covariance (ANCOVA) was performed, adjusting for age, sex, BMI, hypertension, diabetes, eGFR, and statin use. After covariate adjustment, the serum concentrations of SEPP1, CILP, and FRZB remained significantly different between the ICM and control groups (adjusted *p* < 0.05), confirming that the biomarker differences remained statistically significant after adjustment for the measured covariates.

**Table 3:** Baseline clinical characteristics of the ELISA cohort.

Characteristic	Controls (*n* = 45)	ICM Patients (*n* = 45)	*p* value
Demographics			
Age (years), mean ± SD	61.8 ± 7.5	63.4 ± 8.2	0.342
Male sex, *n* (%)	29 (64.4%)	32 (71.1%)	0.498
BMI (kg/m^2^), mean ± SD	24.8 ± 3.1	25.6 ± 3.4	0.250
Comorbidities & Risk Factors			
Hypertension, *n* (%)	18 (40.0%)	28 (62.2%)	0.035
Diabetes mellitus, *n* (%)	9 (20.0%)	15 (33.3%)	0.155
Smoking history, *n* (%)	15 (33.3%)	21 (46.7%)	0.198
Laboratory Findings			
eGFR (mL/min/1.73m^2^), mean ± SD	82.3 ± 11.8	76.5 ± 14.2	0.039
LDL-C (mmol/L), mean ± SD	2.85 ± 0.62	2.12 ± 0.55	<0.001
NT-proBNP (pg/mL), median (IQR)	65 (35–98)	1850 (890–3420)	<0.001
Cardiac Function			
LVEF (%), mean ± SD	64.2 ± 4.3	38.4 ± 6.5	<0.001
NYHA Class II/III/IV, n	N/A	12/25/8	-
Medications & History			
Prior MI/PCI/CABG, *n* (%)	0 (0%)	38 (84.4%)	<0.001
Statin use, *n* (%)	12 (26.7%)	39 (86.7%)	<0.001
ACEI/ARB/ARNI use, *n* (%)	8 (17.8%)	35 (77.8%)	<0.001
Beta-blocker use, *n* (%)	5 (11.1%)	36 (80.0%)	<0.001

(Categorical variables were compared using the Chi-square test; continuous variables were compared using Student’s t-test or Mann–Whitney U test depending on normality. eGFR, estimated glomerular filtration rate; LDL-C, low-density lipoprotein cholesterol; LVEF, left ventricular ejection fraction.).

### 3.8 Immune infiltration analysis

**Figure 8:**
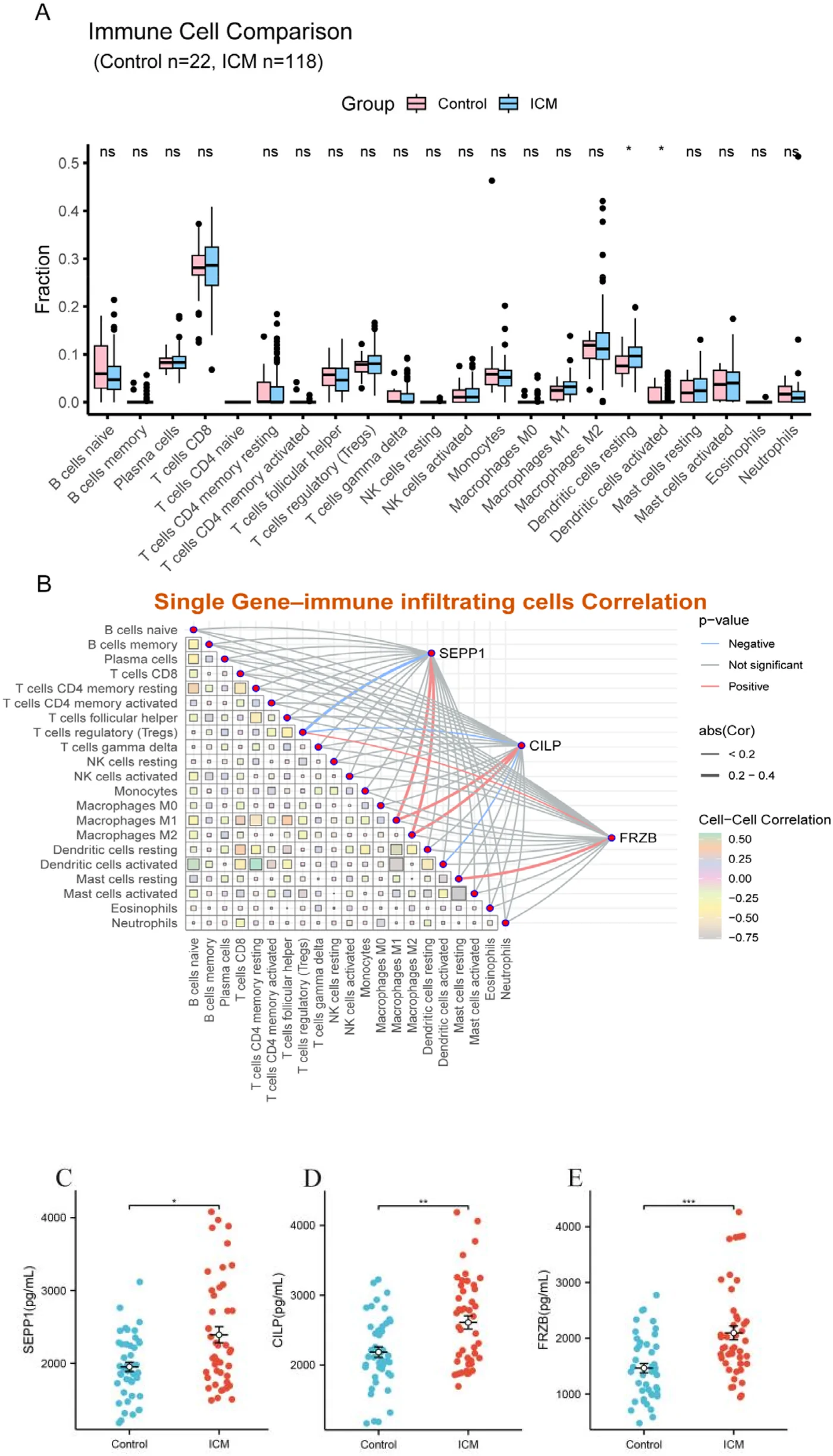
Analysis of immune cell infiltration in ICM. **(A)** Comparative analysis using vioplot, showing the percentage of 22 different types of immune cells in the ICM group and the normal group. **(B)** Correlation analysis of 3 ICM-related biomarkers with various immune cells. **(C–E)** Serum protein concentrations of the key targets measured in peripheral blood from two groups using ELISA.*p < 0.05, **p < 0.01, ***p < 0.001.

The CIBERSORT algorithm calculated the infiltration proportions of 22 immune cell types in each sample. The results indicated nominal trends suggesting an increase in the proportion of resting dendritic cells and a decrease in activated dendritic cells in ICM samples compared to normal samples (Figure 8A). However, after applying Benjamini-Hochberg multiple-comparison correction, no immune cell fractions showed statistically significant differences (all adjusted *p* > 0.05). We further analyzed the relationships between the three core diagnostic genes and immune cell infiltration. SEPP1 showed positive correlations with M1 and M2 macrophages, and negative correlations with regulatory T cells (Tregs) and monocytes. CILP was positively correlated with M1 and M2 macrophages and negatively correlated with Tregs. FRZB exhibited positive correlations with Tregs and resting mast cells. However, these associations did not retain significance after FDR correction. Thus, these computational findings are strictly exploratory and hypothesis-generating, suggesting potential associations rather than direct mechanistic regulation of immune cells (Figure 8B). Subsequently, we measured the serum protein concentrations of the three core diagnostic targets using ELISA analysis, and observed that the protein levels of SEPP1, CILP and FRZB were significantly higher in ICM patients compared to those in the control group (Figures 8C–E). This provides preliminary serological evidence supporting their diagnostic potential, though their exact biological roles in ICM warrant further experimental investigation.

## 4 Discussion

ICM represents a significant health concern, characterized by heart structure and function abnormalities that arise from insufficient coronary blood flow. This condition often leads to severe clinical outcomes, including heart failure and sudden cardiac death, posing substantial burdens on patients and healthcare systems alike (34). The underlying pathophysiology of ICM is primarily linked to coronary artery disease, which diminishes myocardial perfusion and results in ischemic damage (35). Current diagnostic approaches heavily rely on clinical imaging techniques and serum biomarkers, yet their efficacy in potential diagnosis remains limited. The lack of robust molecular-level diagnostic tools hampers timely intervention and personalized treatment strategies, thereby underscoring the urgent need for a deeper understanding of the molecular mechanisms that govern ICM and the development of precise diagnostic models (36, 37).

This study aims to address the critical gap in ICM diagnostics by focusing on the gene expression profiles associated with the disease. Notably, we explore DEGs and their regulatory roles in the pathogenesis of ICM. Previous literature has identified several genes implicated in the pathological processes of ICM; however, a comprehensive analysis integrating gene networks and advanced machine learning methods for biomarker discovery remains scarce (38–40). Employing a combination of differential gene expression analysis and WGCNA, we further delineate biologically relevant gene modules. By harnessing multiple machine learning algorithms, we aim to identify key genes that are closely associated with ICM, thereby innovating diagnostic potential at the molecular level. This research not only sheds light on the intricate gene regulatory networks involved in ICM but also aims to contribute to the identification of novel biomarkers that could facilitate potential diagnosis and targeted therapeutic strategies for this debilitating condition.

The identification of DEGs related to ICM has revealed a significant array of potential biomarkers that could enhance the understanding of the disease mechanism and facilitate potential diagnosis. In this study, we successfully identified 501 DEGs, with 288 upregulated and 213 downregulated genes. The use of WGCNA allowed for the identification of nine gene modules, with the MEcyan module showing a strong positive correlation with ICM (correlation coefficient of 0.66, *p* = 1.2e-18) and comprising 13 specific genes. Notably, 12 genes were found to overlap between the DEGs and the key module genes, presenting a cohesive insight into the genetic landscape of ICM. This finding underscores the importance of understanding the expression patterns of these DEGs in relation to the pathophysiological progress of ICM and highlights their potential as biomarkers for diagnosis and therapeutic targets. Previous research has indicated that alterations in gene expression are critical to the progression of cardiovascular diseases, and our results align with these findings by elucidating the molecular underpinnings of ICM (41–45).

Further, the functional enrichment analysis performed on the identified DEGs revealed significant associations with biological processes including complement activation, TGF-*β* signaling, and extracellular matrix (ECM) organization. These pathways are known to play crucial roles in cardiac repair and remodeling processes following ischemic injury. Specifically, the involvement of the complement system in ICM suggests that immune activation may contribute to myocardial damage and remodeling (46, 47). The TGF-*β* pathway, recognized for its role in fibrosis, may further elucidate mechanisms underlying cardiac remodeling and dysfunction in ICM (48, 49). The ECM, being a critical structural component of the myocardium, is essential for maintaining cardiac function and integrity; its reorganization during ICM contributes to heart failure (50, 51). Therefore, the identified pathways not only provide insights into the molecular mechanisms of ICM but also present potential therapeutic targets for intervention.

The machine learning approaches employed in this study to develop diagnostic models marked a significant advancement in the identification of key genes associated with ICM. Among the 12 algorithms tested, the ADA (Adaptive Boosting) model emerged as the most effective, achieving an area under the curve (AUC) of 0.976, which indicates a high exploratory diagnostic performance. The three key genes identified—SEPP1, CILP, and FRZB—demonstrated consistent diagnostic potential across training and validation datasets (AUC > 0.75 in training, AUC > 0.95 in external validation). This innovative approach not only enhances the accuracy of ICM diagnostics but also suggests that machine learning algorithms can effectively sift through complex biological data to identify significant biomarkers that may otherwise be overlooked.

The development of a nomogram based on these three genes further strengthens the clinical potential of our findings. The calibration curve illustrated a high degree of accuracy in risk prediction, while DCA suggested the model's potential net benefit in the computational datasets. Pending further validation in large-scale prospective clinical trials, implementing such a diagnostic tool could potentially aid healthcare providers in the diagnosis and management of ICM, ultimately leading to better patient outcomes (52).

In addition to the identified biomarkers, our exploratory analysis of immune cell infiltration revealed nominal trends in dendritic cell proportions and potential associations between our core genes and specific immune subsets (53). However, these computational findings did not retain statistical significance after rigorous multiple-comparison correction (all adjusted *p* > 0.05). Therefore, these associations are strictly hypothesis-generating and do not establish any mechanistic modulation of the immune response. Future targeted experimental studies are warranted to evaluate their exact roles in the immune microenvironment and any potential implications for immunomodulatory therapies in the treatment of ICM (54).

The translation of these myocardial transcriptomic signatures to circulating serological proteins likely involves dual pathological mechanisms. Because SEPP1 and FRZB are inherently secreted proteins functioning in antioxidant defense and signaling modulation, their upregulation during ischemia suggests active secretion into the bloodstream as stress-induced injury markers (44, 53). Conversely, CILP is a structural extracellular matrix (ECM) protein; its elevated serum levels likely reflect passive leakage following myocardial necrosis and fibrotic ECM degradation (43).

In conclusion, this comprehensive study elucidates critical genetic and immunological factors involved in ischemic cardiomyopathy. The identification of key biomarkers through differential gene expression analysis, functional enrichment, and machine learning-based model development lays the groundwork for improved diagnostic strategies. Furthermore, understanding the immune landscape associated with these biomarkers may open new avenues for therapeutic interventions aimed at modulating the immune response in ICM, thereby addressing the urgent need for effective treatments in this patient population. Future research should focus on validating these findings in clinical settings and exploring the therapeutic implications of the identified biomarkers and pathways.

The current study presents several limitations that warrant careful consideration. Primarily, our analysis is based on publicly available transcriptomic datasets, which inherently lack direct experimental validation of gene functions. This limitation restricts our ability to provide conclusive evidence regarding the biological roles of the identified genes in ICM. Furthermore, the relatively small sample size, particularly in the external validation cohort, may undermine the generalizability and robustness of the developed diagnostic model. While we employed rigorous statistical methodologies to mitigate potential biases, such as batch effects, the presence of confounding factors in different datasets could still influence the observed results. Additionally, detailed clinical metadata (e.g., NYHA class, LVEF, and ethnicity) were unavailable in these retrospective datasets, necessitating future large-scale prospective studies to evaluate their specific impacts. Such integrative analyses could potentially unveil novel insights into the disease and enhance the accuracy of diagnostic and therapeutic strategies. While our diagnostic model strategically prioritized upregulated genes (derived from the positively correlated cyan module) to maximize signal-to-noise ratios for serological detection, negatively correlated networks (such as the green module) should not be overlooked. These downregulated genes likely represent compromised cardioprotective pathways and serve as complementary targets for future mechanistic investigations.

Additionally, from a methodological perspective, performing DEG and WGCNA feature pre-selection on the entire primary dataset prior to cross-validation introduces theoretical information leakage. Consequently, the reported internal AUC cannot be regarded as a leakage-free estimate of model generalizability, and the machine-learning performance must be interpreted as exploratory and potentially optimistic. To mitigate this limitation, our study relies on the completely untouched, independent external cohort (GSE26887) and real-world serological ELISA experiments to objectively confirm the true generalizability of the identified biomarkers.

Furthermore, while our core diagnostic genes achieved high univariate AUC values (>0.95) in the external dataset (GSE26887), these results must be interpreted with extreme caution. This “performance inversion” relative to the training set is likely a statistical artifact driven by the small sample size (only 5 normal controls), which inevitably produces highly unstable AUC estimates with wide confidence intervals. Consequently, this external assessment cannot fully compensate for the information leakage present in the internal machine-learning pipeline. Additionally, clinical heterogeneity, such as the diabetic comorbidities present in the GSE26887 cohort, may have synergistically amplified the differential expression of these markers compared to the general ICM cohort. Therefore, these external validations should be viewed merely as preliminary estimates rather than definitive clinical evidence.

Finally, it is critical to note that our ELISA experiment measured circulating protein concentrations, which provides preliminary protein-level evidence but is not equivalent to an independent validation of the transcriptomic-derived multivariable diagnostic model. Due to the limited sample size of the current clinical cohort (*n* = 90), we did not construct a combined serum-based multivariable diagnostic model or evaluate its decision-curve analysis (DCA) and calibration in the real-world setting. Future studies involving large-scale, prospective cohorts are warranted to develop a robust serum-protein diagnostic model and to directly compare its sensitivity and specificity with conventional clinical biomarkers such as NT-proBNP or troponin.

## Data Availability

The original contributions presented in the study are included in the article/Supplementary Material, further inquiries can be directed to the corresponding author.
